# Good syndrome and other causes of cytomegalovirus retinitis in HIV-negative patients—case report and comprehensive review of the literature

**DOI:** 10.1186/s12348-016-0070-7

**Published:** 2016-01-25

**Authors:** Kenneth M. Downes, Dariusz Tarasewicz, Laurie J. Weisberg, Emmett T. Cunningham

**Affiliations:** The Department of Ophthalmology, California Pacific Medical Center, 2340 Clay Street 5th, San Francisco, CA 94115 USA; The Department of Ophthalmology, Kaiser Permanente Medical Center, South San Francisco, CA USA; The Department of Hematology/Oncology, Kaiser Permanente South San Francisco Medical Center, South San Francisco, CA USA; The Department of Ophthalmology, Stanford University School of Medicine, Stanford, CA USA; The Francis I. Proctor Foundation, UCSF School of Medicine, San Francisco, CA USA; West Coast Retina Medical Group, San Francisco, CA USA

**Keywords:** Herpetic retinitis, Immunosuppression, Thymoma, Uveitis, Good syndrome

## Abstract

We describe a 65-year-old Thai woman who developed cytomegalovirus retinitis (CMVR) in the setting of Good syndrome—a rare, acquired partial immune deficiency caused by thymoma. The patient subsequently developed vitritis with cystoid macular edema (CME) similar to immune recovery uveitis (IRU) despite control of the retinitis with antiviral agents. A comprehensive review of the literature through December, 2014, identified an additional 279 eyes of 208 patients with CMVR in the absence of human immunodeficiency virus (HIV) infection. Including our newly reported case, 9 of the 208 patients (4.3 %) had Good syndrome. Twenty-one of the 208 patients (10.1 %) had CMVR related to intraocular or periocular corticosteroid administration. The remaining 178 patients (85.6 %) acquired CMVR from other causes. Within the subset of patients who did not have Good syndrome or did not acquire CMVR followed by intraocular or periocular corticosteroid administration, there were many other factors contributing to a decline in immune function. The most common included age over 60 years (33.1 %), an underlying malignancy (28.7 %), a systemic autoimmune disorder requiring systemic immunosuppression (19.1 %), organ (15.2 %) or bone marrow (16.3 %) transplantation requiring systemic immunosuppression, and diabetes mellitus (6.1 %). Only 4.5 % of the patients had no identifiable contributor to a decline in immune function. While the clinical features of CMVR are generally similar in HIV-negative and HIV-positive patients, the rates of moderate to severe intraocular inflammation and of occlusive retinal vasculitis appear to be higher in HIV-negative patients.

**Electronic supplementary material:**

The online version of this article (doi:10.1186/s12348-016-0070-7) contains supplementary material, which is available to authorized users.

## Review

### Introduction

Thymoma is an uncommon and slow-growing neoplasm that accounts for approximately 20 to 30 % of mediastinal masses in adults and 1 % in children [1]. Thymic tumors not only usually present with respiratory symptoms due to compression of the upper airways and/or superior vena cava syndrome but also can produce paraneoplastic or parathymic syndromes [1–4], and the most common of which are myasthenia gravis (MG), pure red cell aplasia (PRCA), and acquired partial immune deficiency or Good syndrome [5, 6].

Good syndrome was first described by the American hematologist-oncologist Dr. Robert Good in 1956 [7]. Good noted a direct relationship between the presence of thymoma and hypogammaglobulinemia causing immunosuppression in those patients. Good syndrome typically occurs in middle-aged adults and is associated most commonly with recurrent sinus and pulmonary infections, cytomegalovirus (CMV) disease (most often retinitis), fungal infections, pure red cell aplasia, and myasthenia gravis [8]. While hypogammaglobulinemia in the setting of thymoma defines Good syndrome, other, often partial, immune deficiencies have also been described, including decreased T cell function [9].

We describe a 65-year-old woman who developed CMV retinitis (CMVR) in the setting of Good syndrome. The patient subsequently developed vitritis with cystoid macular edema (CME) despite control of the retinitis with antiviral agents. Cases of CMVR in human immunodeficiency virus (HIV)-negative patients, including those with Good syndrome, identified in PubMed through December, 2014, were reviewed and are summarized. Search terms included “Cytomegalovirus AND eye” and “cytomegalovirus retinitis.” Additional publications were identified by reviewing collected references.

### Case report

A 65-year-old Thai woman presented for evaluation of suddenly decreased vision with floaters in her right eye. Past ocular history was unremarkable. Past medical history was notable for PRCA diagnosed 2 years prior to presentation and for which she was treated for four months with erythropoietin and systemic corticosteroids. She also had two prior episodes of oropharyngeal candidiasis, which were treated successfully. There was no history of recent or current corticosteroid use.

**Fig. 1:**
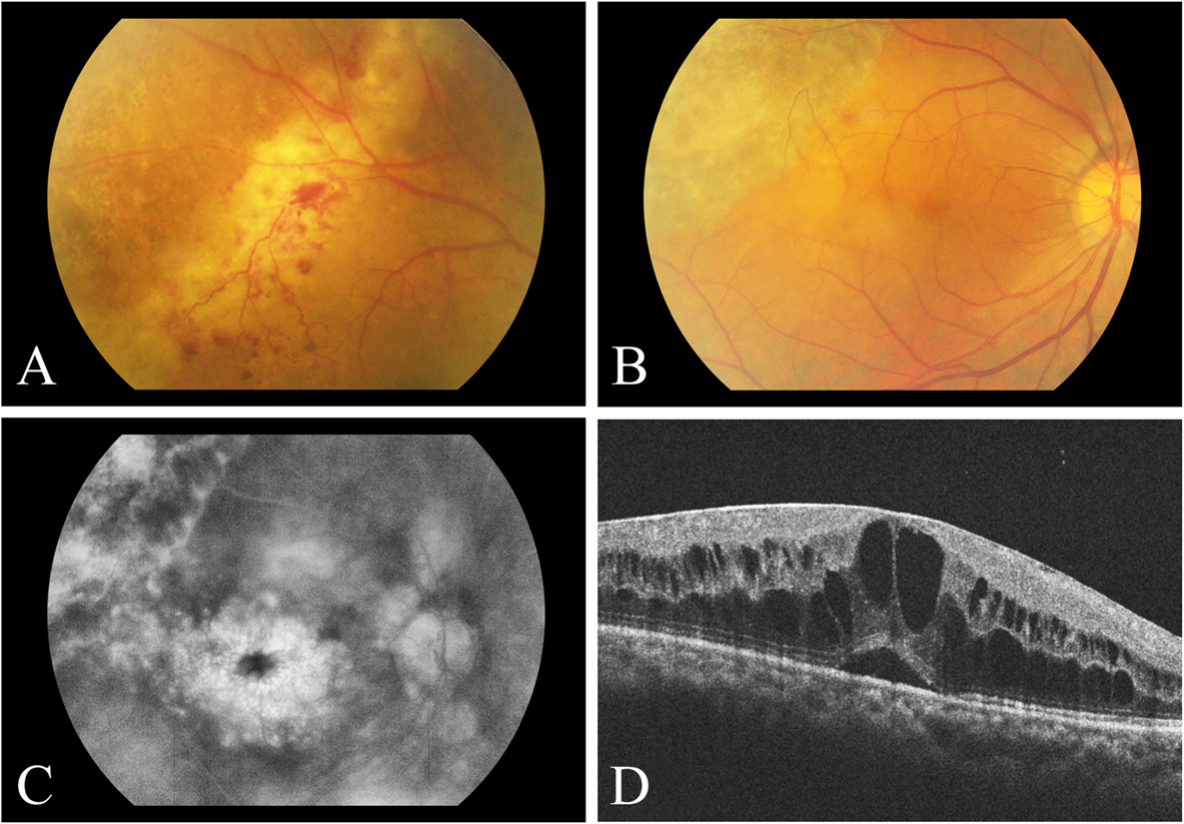
Color photograph of the patient’s right eye showing the active edge of cytomegalovirus retinitis (**a**), which became inactive following treatment with an intravitreal injection of 2 mg of ganciclovir followed by high-dose oral valaciclovir, 2 g three times daily (**b**). Fluorescein angiography (**c**) and SD-OCT imaging (**d**) showed the development of cystoid macular edema consistent with the diagnosis of immune recovery uveitis

The best-corrected vision was 20/100 on the right eye and 20/25 on the left eye. Intraocular pressure (IOP) was 15 mmHg bilaterally. No afferent pupillary defect was noted. Anterior segment examination of the right eye showed trace anterior chamber cell but was otherwise normal. Anterior segment examination on the left eye was unremarkable. Posterior segment examination on the right showed moderate vitreous inflammation and an advancing edge of necrotizing retinitis associated with scattered intraretinal hemorrhages and retinal vascular telangiectasis (Fig. 1a). Posterior segment examination of the left eye was unremarkable. The patient was diagnosed clinically with viral retinitis, an anterior chamber paracentesis was performed for viral DNA testing, a laser barrier was applied immediately posterior to the area of active retinitis, and the patient was given an intravitreal injection of 2 mg of ganciclovir followed by treatment with high-dose oral valaciclovir, 2 g three times daily. Analysis of the anterior chamber paracentesis was positive for CMV DNA, and the patient was switched from valaciclovir to valganciclovir, which resulted in resolution of the area of retinitis.

The patient subsequently underwent HIV and syphilis testing, colonoscopy, chest X-ray, bone marrow biopsy, and abdominal CT, all of which were negative. Testing of immune function revealed a markedly decrease total B cell (CD19) cell count and panhypogammaglobulinemia (Table 1).

**Table 1:** Immunologic profile over time of currently reported case of cytomegalovirus retinitis in the setting of thymoma (Good’s syndrome)

Immunologic profile	At time of retinitis	At time of CME diagnosis and 5 months after thymoma resection	6 months after CME and 11 months after thymoma resection	Reference range
T cells CD3	1079 (70 %)	N/A	1284 (81 %)	672–2638 cells/mL (54–83 %)
T helper cells CD4	480 (31 %)	N/A	437 (28 %)	292–1366 cells/mL (23.1–51.0 %)
Cytotoxic T cells CD 8	540 (35 %)	N/A	817 (51 %)	240–1028 cells/mL (17.9–47.5 %)
CD4 to CD8 ratio	0.88	N/A	0.53	0.6–2.5
B cells CD19	40 (3 %)	N/A	26 (2 %)	82–560 cells/mL (5.1–20.8 %)
Natural killer cells CD16/56	413 (27 %)	N/A	277 (17 %)	130–938 cells/mL (7.1–38.0 %)
Immunoglobulin G	2.24	1.91	N/A	8–18 (g/L)
Immunoglobulin M	<0.06	<0.06	N/A	0.5–2.2 (g/L)
Immunoglobulin A	<0.04	0.6	N/A	1.1–5.6 (g/L)

*Abbreviations*: *CME* cystoid macular edema, *CMV* cytomegalovirus, *NR* not reported, *IgG* immunoglobulin G, *IgA* immunoglobulin A, *IgM* immunoglobulin M, *CD* cluster designation, *NK* natural killer, *N/A* not available

**Fig. 2:**
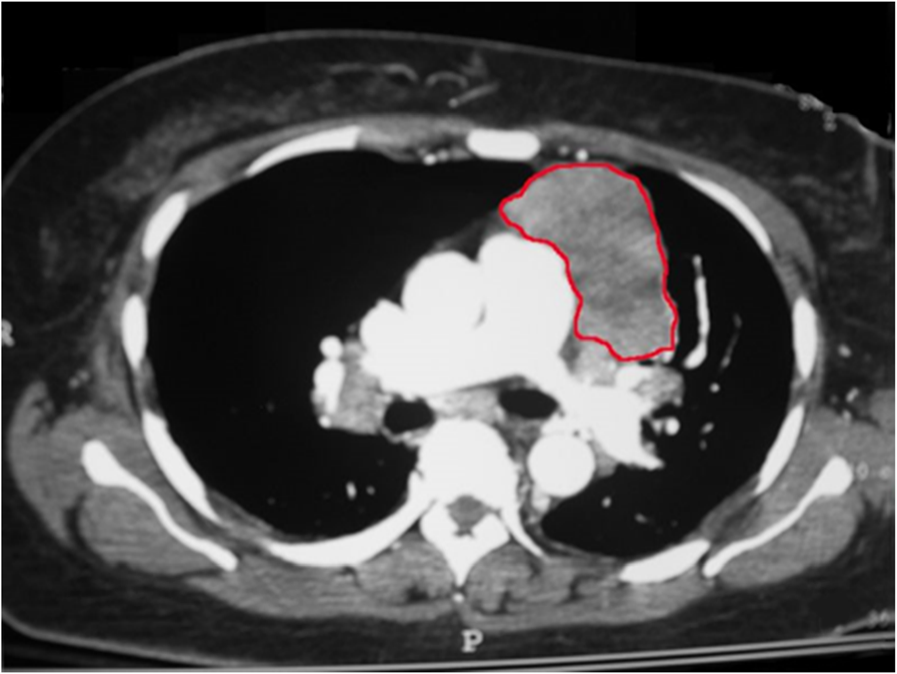
Chest CT showing a large mediastinal mass outlined in *red* and found subsequently to be a thymoma

Within 1 month of the diagnosis of CMVR, the patient was hospitalized for acute pneumonia. During this hospitalization, a mediastinal mass was discovered on chest X-ray and evaluated further by chest CT (Fig. 2). Computer tomography guided thymus biopsy and subsequent thymectomy were performed, revealing histological changes consistent with thymoma that lead to the diagnosis of Good syndrome. Immunoglobulin A, G, and M levels remained low at the last testing 5 months following removal of the thymus. The patient then returned with worsening vision in the eye with CMVR while on maintenance valganciclovir therapy, 450 mg twice daily.

The best-corrected vision was 20/125 on the right eye and 20/25 on the left eye. Intraocular pressure was normal. No afferent pupillary defect was noted. Anterior segment examination on the right showed several stellate keratic precipitates on the corneal endothelium, one cell per high powered field in the anterior chamber, and occasional anterior vitreous cells. Anterior segment examination on the left was unremarkable. Posterior segment examination on the right showed mild to moderate vitreous inflammation, a posterior vitreous detachment, a large area of inactive retinal necrosis (Fig. 1b), continuous laser barrier scars immediately posterior to the area of retinitis, and loss of the foveal light reflex suggestive of CME. Posterior segment examination on the left was unremarkable. Fluorescein angiography confirmed the presence of severe CME on the right (Fig. 1c). Spectral domain optical coherence tomography (SD-OCT) imaging showed marked CME on the right (Fig. 1d) with a central macular thickness of 743 um. SD-OCT imaging of the left fovea revealed a normal contour with no evidence of subretinal or intraretinal fluid. The patient was treated with topical difluprednate four times daily for 1 month. The CME persisted, the difluprednate was stopped, and the patient was given two injections of 1.25 mg of intravitreal bevacizumab, 1 month apart. The CME failed to respond, and so the patient was given an intravitreal injection of triamcinolone acetonide, 2.0 mg, following which the CME resolved and vision improved to 20/80 in the affected eye. The CME subsequently recurred and the vision decreased to 20/100, but the patient refused further treatment. The retinitis remained inactive.

### Comprehensive literature review

We describe a patient who developed CMVR in the setting of Good syndrome, a rare occurrence reported in eight previous patients to date (Tables 2 and 3) [10–16]. Including our patient, reported ages of the nine patients ranged from 48 to 68 years, with both a mean and median of 56 years. Women constituted just over half of the reported patients (55.5 %), with retinitis occurring unilaterally in all but one patient (88.9 %) and involving zone 1 in nearly two thirds of the affected eyes (62.5 %). When reported, anterior chamber inflammation was present in 62.5 % of cases; vitritis was present in 88.8 % of cases and was reported to be moderate to severe in five cases (55.5 %). The diagnosis was confirmed in all but one patient (89.9 %) by polymerase chain reaction (PCR)-based testing of intraocular fluids, and all cases responded to antiviral therapy, which was administered both intravitreally and systemically in six of nine (66.6 %) patients. While the CMVR in our patient occurred 1 month prior to the identification of thymoma, a thymic tumor was identified prior to the development of CMVR in the other eight patients, with a time ranging from 1 month to just over 6 years prior to the occurrence of retinitis. Visual acuity at the initial CMVR diagnosis was between 20/40 and 20/200 in 77.8 and worse than 20/200 in 22.2 % of eyes, whereas visual acuity at last follow-up (median 6 months; range 1.5–7 months) was between 20/40 and 20/200 in 55.5 and worse than 20/200 in 44.4 % of eyes. Other common opportunistic infections reported in these nine patients with Good syndrome and CMVR included respiratory infections (77.8 %), non-ocular CMV (22.2 %), and herpes zoster dermatitis (33.3 %), whereas other autoimmune diseases (Table 3) included MG (25.0 %) and PRCA (22.2 %).

**Table 2:** Summary of the current and previously reported cases of cytomegalovirus (CMV) retinitis in the setting of immunodeficiency associated with thymoma (Good syndrome)

	Author (year)	Age (years)	Gender	Unilateral (U) or bilateral (BL)	Timing of CMV retinitis relative to thymoma diagnosis (months)	Associated opportunistic infections^a^	Zone (involved)^b^	Retinitis treatment^c^	CMV testing	Vision when retinitis was first diagnosed	Follow-up (months)	Vision at the last visit
Previously published cases	Ho et al. (2010) [10]	68	M	U	75 months after thymoma	Recurrent pneumonia; disseminated CMV; CMV colitis	Zone I	IV ganciclovir and then PO valganciclovir	Lung biopsy	20/200	1	20/100
	Mateo-Montoya et al. (2010) [11]	57	M	U	NR. “Long time after thymoma”	Recurrent pneumonia; *Camplyobacter* sepsis	Zone I	IVT ganciclovir, IVT foscarnet, and IV ganciclovir then PO valganciclovir	Aqueous PCR; vitreous PCR	20/100	6	20/40
	Park et al. (2009) [12]	56	M	BL	3 months after thymoma	NR	Zone I and zone II	IVT ganciclovir and IV ganciclovir, then PO valganciclovir	Aqueous PCR; serum IgG	20/800 OD, 20/125 OS	6	CF at 30 cm OD, NLP OS
	Sen et al. (2005)1 [13]	48	M	U	60 months after thymoma	*Pneumocystis jiroveci* pneumonia; history of retinitis and optic neuropathy	Zone I	IVT ganciclovir and IVT ganciclovir implant (Vitrasert)	Aqueous PCR	20/200	7	20/200
	Wan et al. (2012) [14]	51	F	U	60 months after thymoma	Recurrent sinopulomary infections; CMV enterocolitis	Zone II	PO valganciclovir then IVT ganciclovir weekly	Vitreous PCR	20/40	6	20/50 then subsequently to CF due to the development of autoimmune retinopathy
	Yong et al. (2008) [15]	50	F	U	6 months after thymoma	Herpes zoster (T10 dermatome)	NR	IV ganciclovir, then PO valganciclovir	Vitreous PCR	NR	2	NLP
	Assi et al. (2002) [16] case 1	45	F	U	24 months after thymoma	Recurrent pneumonia; zoster dermatitis	Zone II and zone III	IV valaciclovir, then IVT ganciclovir implant	Vitreous PCR	20/40	6 weeks	NR
	Assi et al. (2002) [16] case 2	65	F	U	24 months after thymoma	Recurrent pneumonia	Zone I	IVT foscarnet then PO ganciclovir	Vitreous PCR	HM	NR	HM
Current case	Downes, et al. (2016)	65	F	U	1 month before thymoma	Oropharyngeal candidiasis; *Candida esophagitis*; pneumonia	Zone II and zone III	IVT ganciclovir, then PO valganciclovir	Aqueous PCR	20/100	7	20/80
Summary	Total *n* = 9	Mean: 56 years	Male: 4/9 (44.4 %)	8/9 (88.9 %) unilateral	Retinitis diagnosed after thymoma: 8/9 (88.9 %)	Respiratory infections: 7/9 (77.8 %)	Zone I: 5/8 reported (62.5 %)	Intravitreal therapy alone: 1/9 (11.1 %)	Positive aqueous PCR: 4/9 (44.4 %)	Acuity better than 20/40: 0/9 eyes (0.0 %)	Mean = 4.56 months	Acuity better than 20/40: 0/9 eyes (0.0 %)
		Median: 56 years	Female: 5/9 (55.5 %)		Mean = 31.4 months after thymoma	Non-ocular CMV: 2/9 (22.2 %)	Zone II: 4/8 reported (50 %)	Systemic therapy alone: 2/9 (22.2 %)	Positive vitreous PCR: 5/9 (55.5 %)	Acuity between 20/40 and 20/200: 7/9 eyes (77.8 %)	Median = 6 months	Acuity between 20/40 and 20/200: 5/9 eyes (55.5 %)
		Range: 48–68 years	Male to female ratio 0.8:1		Median = 24 months after thymoma	Other opportunistic infections: 3/9 (33.3 %)	Zone III: 2/8 reported (25 %)	Combo intravitreal and systemic therapy: 6/9 (66.6 %)	Confirmed by other means: 1/9 (11.1 %)	Acuity worse than 20/200: 2/9 eyes (22.2 %)	Range = 1.5–7 months	Acuity worse than 20/200: 4/9 eyes (44.4 %)
					Range = 75 months after to 1 month before							

Abbreviations: *IgG* immunoglobulin G, *M* male, *F* female, *U* unilateral, *BL* bilateral, *CMV* cytomegalovirus, *NR* not reported, *CF* count fingers, *NLP* no light perception, *LP* light perception, *HM* hand motion, *IV* intravenous, *PO per oral*, *IVT* intravitreal, *PCR* polymerase chain reaction

^a^All patients were tested for HIV and found to be negative

^b^Zone definitions are as follows: zone I defined as macula or optic nerve involvement; zone II defined as mid-periphery; and zone 3 defined as outer periphery. Zone definitions referenced in this paper: Cunningham ET Jr, Hubbard LD, Danis RP, Holland GN. Proportionate topographic areas of retinal zones 1, 2, and 3 for use in describing infectious retinitis. Arch Ophthalmol. 2011;129(11):1507–8 [115]

^c^Dosing with each modality varied widely across studies

**Table 3:** Summary of autoimmune conditions and immunologic parameters in current and previously reported cases of cytomegalovirus (CMV) retinitis in the setting of immunodeficiency associated with thymoma (Good syndrome)

	Author (year)	Myasthenia gravis	Pure red cell aplasia	Other conditions encountered	Lymphopenia^a^	Low CD3+ T cells (<672 cells/mL or <54 %)	Low CD4+ count (<360/μL or <36 %)	A low CD8+ count (<240 cells/μL)	Low CD4+/CD8+ ratio (<0.6)	Low NK cells (<130 cells/mL or <7.1 %)	Hypogammaglobulinemia IgG (IgG < 8 g/L)	Hypogammaglobulinemia IgM (IgM <0.5 g/L)	Hypogammaglobulinemia IgA (IgA < 1.1 g/L)	Panhypogammaglobulinemia (I gG < 8 g/L, IgM <0.5 g/L, IgA < 1.1 g/L, or total Ig < 9.6 g/L)
Previously published cases	Ho et al. (2010) [10]	−	−	None	+	+	+	−	+	+	+	+	+	+
	Mateo-Montoya et al. (2010) [11]	+	−	None	NR	NR	+	NR	NR	NR	NR	NR	NR	+
	Park et al. (2009) [12]	−	−	None	+	NR	NR	NR	NR	NR	+	+	+	+
	Sen et al. (2005) [13]	−	−	?neurosensory hearing loss and optic neuropathy	NR	NR	+	NR	+	NR	+	+	+	+
	Wan et al. (2012) [14]	NR	+	Autoimmune retinopathy (many years after diagnosis of retinitis)	NR	NR	NR	NR	NR	NR	NR	NR	NR	NR
	Yong et al. (2008) [15]	−	−	None	+	−	+	−	+	NR	+	+	+	+
	Assi et al. (2002) [16] case 1	+	−	None	−	NR	+	NR	+	NR	−	−	+	−
	Assi et al. (2002) [16] case 2	−	−	None	NR	NR	NR	−	+	NR	+	+	+	+
Current case	Downes, et al. (2013)	−	+	None	−	−	−	−	−	−	+	+	+	+
Summary	Total *n* = 9	+2/8 reported (25.0 %)	+2/9 cases (22.2 %)	No other definite autoimmune conditions encountered at the time of diagnosis of retinitis	+3/5 reported (60.0 %)	+1/3 reported (33.3 %)	+5/6 reported (83.3 %)	+0/4 reported (0.0 %)	+5/6 reported (83.3 %)	+1/2 reported (50.0 %)	+7/8 reported hypo IgG (87.5 %)	+7/8 reported hypo IgM (87.5 %)	+8/8 reported hypo IgA (87.5 %)	+7/8 reported panhypogammaglobulins (87.5 %)

*Abbreviations*: *CMV* cytomegalovirus, *NR* not reported, *IgG* immunoglobulin G, *IgA* immunoglobulin A, *IgM* immunoglobulin M, *CD* cluster designation, *NK* natural killer

^a^Based on each individual lab standards and if reported by authors

Although the retinitis in our patient responded promptly to intravitreal and systemic antiviral agents, the patient subsequently developed vitritis and CME of the type seen in patients with immune recovery uveitis (IRU) despite the fact that her total CD4+ T cell count was normal both before and after the occurrence of retinitis. In contrast, the total B cell count and immunoglobulin levels where low both before and after thymectomy. While hypogammaglobulinemia is required to diagnose Good syndrome and has been observed in all reported cases to date, including our patient, it is noteworthy that the total CD4+ T cell count was somewhat decreased in five of the eight previously reported cases with Good syndrome and CMVR (Table 3), indicating that partial CD4+ T cell depletion does occur in patients with Good syndrome and suggesting the possibility that selective loss of CMV-targeting CD4+ T cells may have occurred in our patient, facilitating the development of retinitis. To our knowledge, an IRU-like syndrome has not been reported previously following treatment of CMVR in a patient with Good syndrome.

Our review identified a total of 248 eyes of 178 patients previously reported with CMVR in the absence of either HIV infection, Good syndrome, or prior periocular or intraocular corticosteroid injection (Additional file 1: Table S1) [17–86]. Reported ages ranged from 1 week to 84 years, with a mean and median of 45.7 and 48.0 years, respectively. Men outnumbered women approximately two to one (M to F ratio = 1.88:1), and the vast majority (95.5 %) had an identifiable cause of systemic immunosuppression. The most common factors contributing to a decline in immune function included age over 60 years (33.1 %), an underlying malignancy (28.7 %), a systemic autoimmune disorder requiring treatment (19.1 %), organ (15.2 %) or bone marrow (16.3 %) transplantation requiring systemic immunosuppression, and diabetes mellitus (6.1 %). The most commonly reported cancers included leukemia (35 patients; 19.7 %) and lymphoma (14 patients; 7.9 %). Three patients had multiple myeloma (1.7 %). One patient each (0.6 %) had breast cancer and angiocentric immunoblastic lymphadenopathy with dysproteinemia. Several patients had a primary immune deficiency other than Good syndrome, including three patients (1.7 %) with severe combined immunodeficiency, two patients each (1.1 %) with unspecified primary immune deficiency and common variable immune deficiency, and one patient (0.6 %) with idiopathic CD4+ T cell lymphopenia. Among the 26 reported patients less 18 years of age, 9 (34.6 %) had acute lymphoblastic leukemia, 8 (30.8 %) had undergone bone marrow transplantation, 5 (19.2 %) had congenital CMV infection, 3 (11.5 %) had severe combined immunodeficiency and 1 each (3.8 %) had common variable immune deficiency and immunoglobulin 2 (Ig2) deficiency.

The use of systemic immunosuppressive medication was reported in 105 of 160 cases (65.6 %). Seventy-eight of these 105 patients (74.3 %) were on two or more immunosuppressive agents. Corticosteroids were the most common immunosuppressive agent used in 69 patients (65.7 %), followed by cyclophosphamide in 33 patients (31.4 %), azathioprine in 17 patients (16.2 %), vincristine in 16 patients (15.2 %), methotrexate in 15 patients (14.3 %), cyclosporine in 13 patients (12.4 %), tacrolimus in 11 patients (10.5 %), 6-mercaptopurine in 9 patients (8.6 %), mycophenolate mofetil in 8 patients (7.6 %), fludarabine in 7 patients (6.7 %), and adriamycin in 6 patients (5.7 %). Five patients each (4.8 %) used intravenous immunoglobulin G and rituximab. One patient each (1.0 %) was on mitoxantrone, ibritumomab tiuxetan, and hydroxychloroquine. Within the 105 cases reporting medication use, the use of either an antimetabolite or a leukocyte signaling inhibitor as a group (methotrexate, azathioprine, mycophenolate mofetil, tacrolimus, or cyclosporine) was reported in 46.7 % of cases, whereas chemotherapeutic agents as a whole (cyclophosphamide, vincristine, high-dose methotrexate, 6-mercaptopurine, adriamycin, rituximab, fludarabine, mitoxantrone, and ibritumomab tiuxetan) were reported in 48.6 % of cases. Clinically, the retinitis was unilateral in 108 out of 178 cases (60.7 %). Many case reports provided limited clinical information, but the location was either reported or illustrated in 97 out of the 248 eyes (39.1 %) and was found to involve zone 1 in 72 eyes (74.2 %). Additional clinical features were noted in 200 out of 248 eyes (80.6 %). Among these, 200 eyes, or just under one third (29.0 %), were noted to have anterior chamber inflammation, which was described as mild in 18 (9.0 %) and moderate in 13 (6.5 %), and severe in the remaining 27 (13.5 %). Just over one third of the reported eyes (37.5 %) were noted to have vitreous inflammation, among which inflammation was described as mild in 23 (11.5 %), moderate in 18 (9.0 %), and severe in 6 (3.0 %), with the remaining 28 (14.0 %) not quantifying severity. An occlusive vasculitis was noted in 47 eyes (23.5 %). Visual acuity at initial CMVR diagnosis was reported in 179 of 248 eyes (72.2 %). Among these 179 eyes, visual acuity at initial diagnosis was better than 20/40 in 34.1 %, between 20/40 and 20/200 in 39.1 %, and worse than 20/200 in 26.8 % of eyes. The method of diagnostic confirmation of CMVR was reported in 131 of the 178 cases (73.6 %), among which the diagnosis of CMVR was confirmed by PCR-based testing of intraocular fluids in 71.8 %. The retinitis responded to antiviral therapy in all cases. The treatment administered was reported in 126 of the 178 cases (70.8 %). Of these 126 cases, systemic treatment was administered in 45.2 % of patients, whereas both intravitreal and systemic therapy was given in 31.0 % of patients, and intravitreal treatment alone was employed in 23.8 % patients. Visual acuity at the last follow-up visit was reported in 171 eyes (mean 14.2 months; median 6.0 months; range 0 to 216 months) and was better than 20/40 in 30.4 %, between 20/40 and 20/200 in 37.4 %, and worse than 20/200 in 32.2 % of eyes.

Recently, Takakura and colleagues reviewed the literature on patients who developed viral retinitis following intraocular or periocular administration of corticosteroids [87]. Out of a total of 30 reported cases, 21 (70.0 %) developed CMVR (Table 4) [33, 87–100]. These 21 patients constituted 10.1 % of the total of 208 non-HIV-positive patients with CMVR identified in our review. Among the 21 patients with CMVR, reported ages ranged from 30 to 84 years, with a mean and median of 66 and 69 years, respectively, and men outnumbered women two to one (M to F ratio = 2:1). The most common underlying ocular diseases for which corticosteroids were injected included diabetes mellitus (38.0 %), retinal vein occlusion (33.3 %), and uveitic CME (33.3 %), followed by choroidal neovascularization secondary to age-related macular degeneration (9.5 %). In patients with uveitis and CMVR, Behcet’s disease and anterior uveitis comprised two cases each (28.5 %), followed by one case each (14.3 %) of anterior uveitis, Vogt-Koyangi-Harada disease, idiopathic posterior uveitis, and idiopathic panuveitis. The corticosteroid was administered intravitreally in 19 of the 21 eyes (90.5 %). Among these 19 patients who received intravitreal corticosteroids, 8 (38.0 %) were administered between 1.5 and 4 mg, 4 (19.0 %) were administered between 8 and 20 mg, and 1 (4.8 %) was administered 40 mg of triamcinolone acetonide. Two (9.0 %) were implanted with the fluocinolone acetonide intravitreal implant (Retisert®). Among the two patients who received periocular corticosteroids, one (4.8 %) was administered 20 mg triamcinolone acetonide while the other patient (4.8 %) was administered 40 mg. The median time to developing retinitis after corticosteroid administration was 4.3 months with a mean of 4.0 months and a range 7 days to 13 months. Clinically, the retinitis involved zone 1 in 20.0 % of the eyes and was both unilateral and ipsilateral to the injection in all cases. Twelve out of 21 cases (57.1 %) described some clinical features of the retinitis. Within these 12 eyes, 10 (83.3 %) were noted to have anterior chamber inflammation, among which the inflammation was described as mild in one case (8.3 %) and moderate in four cases (33.3 %)—with the remaining seven (58.3 %) not quantifying severity. Over ninety percent of the eyes (91.6 %) were noted to have vitreous inflammation, among which inflammation was described as moderate in four eyes (33.3 %) with the remaining seven (63.6 %) not quantifying the severity of the inflammation. An occlusive vasculitis was noted in seven eyes (58.3 %). Visual acuity at initial CMVR diagnosis was reported in all 21 eyes and was better than 20/40 in 2 eyes (9.5 %), between 20/40 and 20/200 in 11 eyes (52.3 %), and worse than 20/200 in 8 eyes (38.0 %). The diagnosis was confirmed by PCR-based testing of intraocular fluids in 95.2 % of cases, and all cases responded to antiviral therapy, which was administered both intravitreally and systemically in 11 patients (52.3 %). Visual acuity at the last follow-up visit (mean 11.8 months; median 5.5 months; range 1–84 months) was better than 20/40 in 2 eyes (9.5 %), between 20/40 and 20/200 in 8 eyes (38.0 %), and worse than 20/200 in 11 eyes (52.3 %).

**Table 4:** Summary of previously reported HIV-negative cases of cytomegalovirus retinitis following intraocular and periocular corticosteroid injection

Previously published cases	Author (year)	Age (years)	Gender	Unilateral (U) or bilateral (BL)	Indication for corticosteroid	Corticosteroid dose/route	Time from corticosteroid dosing to retinitis (months)	CMV testing	Zone involved^a^	Vision when retinitis was first diagnosed	Retinitis treatment^b^	Follow-up (months)	Vision at the last Visit
	Saidel et al. (2005) [88]	75	M	U	DME	4 mg IVTA	4.0	CMV retinitis (PCR)	Zone I	20/400	IV ganciclovir, then IV valganciclovir, repeated IVT ganciclovir	6	20/400
	Delyfer et al. (2007) [89] Case 1	77	M	U	CNVM/AMD	20 mg IVTA	4.0	CMV retinitis (serology + PCR)	Zone II	CF at 1.8 m	IVT, IV valganciclovir	6	20/200
	Delyfer et al. (2007) [89] Case 2	69	M	U	CRVO/DME	8 mg IVTA three times over a 3-month interval	3 (after the 3rd IVTA)	CMV retinitis (PCR)	Zone I	20/200	IV ganciclovir and IV valganciclovir	3	20/400
	Furukawa et al. (2007) [90]	54	F	U	DME	10 mg IVTA	4.0	CMV retinitis (serology + PCR)	Zone II	1	IV ganciclovir; IVT foscarnet; vitrectomy and silicone oil tamponade	14	0.5
	Hsu et al. (2007) [91]	77	M	U	DME	4 mg IVTA	1.5	CMV retinitis (PCR)	Zone II	3/200	Valganciclovir	1	20/400
	Ufret-Vincenty et al. (2007) [92]	65	M	U	Uveitic CME/Behcet’s disease	FA implant	53 (after the 1st implant), 5 (after the 2nd implant)	CMV retinitis (“clinical diagnosis”)	Zone II	20/50	IVT foscarnet; ganciclovir implant	5	20/40
	Park et al. (2008) [93]	77	F	U	CRVO/CME due to HTN	4 mg IVTA	4.0	CMV retinitis (PCR)	Zone II	LP	IVT ganciclovir	4	HM
	Sekiryu et al. (2008) [94]	63	M	U	BRVO/DME	4 mg IVTA	7.0	CMV retinitis (PCR)	Zone II	0.1	IV ganciclovir, IV valganciclovir	1	0.6
	Babiuch et al. (2010) [95]	77	M	U	Idiopathic iritis	40 mg PST TA	0.25	CMV retinitis (serology + PCR)	Zone II	20/40	Vitrectomy, endolaser; IVT ganciclovir; ganciclovir implant	NR	NR
	Shah et al. (2010) [96]	62	M	U	BRVO/DME	20 mg IVTA *×* 2	6.5	CMV retinitis (PCR)	Zone II	20/400	IV valaciclovir; vitrectomy, endolaser and silicone oil; then IV valganciclovir	NR	NR
	Toyokawa et al. (2010) [97]	83	M	U	CNVM/AMD	20 mg PST TA	3.0	CMV retinitis (PCR)	Zone II	0.3	PO valaciclovir, vitrectomy	5	0.1
	Tugal-Tutkun et al. (2010) [98]	30	M	U	Behcet’s panuveitis	IVTA dose NR	3.5	CMV retinitis (serology + PCR)	Zone I, zone II, zone III	20/200	IVT ganciclovir × 2; IV ganciclovir × 5 weeks; azathioprine changed to interferon alpha 2a	8	20/60
	Vertes et al. (2010) [99]	78	F	U	BRVO/CME	4 mg IVTA	3.0	CMV retinitis (serology + PCR)	Zone II	20/40	IV ganciclovir; PO ganciclovir; IVT ganciclovir; then vitrectomy, endolaser	8	20/25
	Zaborowski et al. (2013) [100]	56	F	U	Idiopathic panuveitis/Uveitic CME	4 mg IVTA	6.0	CMV retinitis (PCR)	Zone II	CF	Azathioprine discontinued; intravitreal ganciclovir twice weekly for 3 weeks (2 mg)	2	NR
	Gupta et al. (2013) [32] case 1	70	F	U	DME	IVTA dose NR	4.0	CMV retinitis (PCR)	Zone II	CF	IVT foscarnet; IV valaciclovir; IVT foscarnet × 2; ganciclovir implant	32	CF
	Gupta et al. (2013) [32] case 2	60	M	U	DME	IVTA NR	6.0	CMV retinitis (PCR)	NR	20/400	intravitreal foscarnet; IV valganciclovir; ganciclovir implant	NR	20/300
	Gupta et al. (2013) [32] case 3	84	F	U	BRVO	IVTA NR	6.0	CMV retinitis (PCR)	Zone II	20/150	IVT foscarnet; IV valganciclovir	19	HM
	Takakura et al. (2013) [87] case 1	66	M	U	VKH with steroid-induced cataracts and ocular hypertension, IVTA given during cataract surgery	4 mg IVTA and ASCTA	1.8	CMV retinitis (PCR)	Zone II	20/200	IVT ganciclovir, PO valganciclovir; methotrexate and low-dose oral prednisone	2	20/70
	Takakura et al. (2013) [87] case 2	37	F	U	Bilateral idiopathic posterior uveitis complicated by CME/retinal vasculitis	FA implant	13.0	CMV retinitis (PCR)	Zone I	20/80	IVT foscarnet; PO valganciclovir	2	20/100
	Takakura et al. (2013) [87] case 3	63	M	U	Granulomatous uveitis with CME	40 mg IVTA × 2	3.0	CMV retinitis (PCR)	Zone II	20/60	IV ganciclovir; PO prednisone; PPV	84	20/200
	Takakura et al. (2013) [87] case 4	72	M	U	BRVO	4 mg IVTA	1.3	CMV retinitis (PCR)	Zone II	20/60	IVT ganciclovir	12	CF
Summary	*N* = 21	Mean, 66.4 years	Male, 14/21 (66.6 %)	21/21 (100 %)	RVO, 7/21 (33.3 %)	1.5–4 mg IVT, 8/21 (38.0 %)	Mean, 4.3 months	Positive aqueous or vitreous PCR, 20/21 (95.2 %)	Zone I, 4/20 reported (20.0 %)	Acuity better than 20/40, 2/21 eyes (9.5 %)	Intravitreal therapy alone, 5/21 (23.8 %)	Mean = 11.8 months	Acuity better than 20/40: 2/21 eyes (9.5 %)
		Median, 69 years	Female, 7/21 (33.3 %)		DME, 8/21 (38.0 %)	8–20 mg, 5/21 (23.8 %)	Median, 4.0 months	Confirmed by other means: 1/21 (4.8 %)	Zone II, 17/20 reported (85.0 %)	Acuity between 20/40 and 20/200, 11/21 eyes (52.3 %)	Systemic therapy alone, 5/21 (23.8 %)	Median = 5.5 months	Acuity between 20/40 and 20/200: 8/21 eyes (38.0 %)
		Range, 30.0–84.0 year	Male to female ratio, 2:1		Uveitic CME, 7/21 (33.3 %)	40 mg, 2/21 (9.0 %)	Range, 0.25–13.0 months		Zone III, 1/20 reported (5.0 %)	Acuity worse than 20/200, 8/21 eyes (38.0 %)	Intravitreal and systemic therapy, 11/21 (52.3 %)	Range = 1–84 months	Acuity worse than 20/200: 11/21 eyes (52.3 %)
					CNVM due to AMD, 2/21 (9.5 %)	FA implant, 2/21 (9.0 %)							
					IRU, 0/21 (0.0 %)	Range, 1.5–40 mg							

Data used from paper done by Takakura et al (2013) currently in peer review Ocular Immunology and Inflammation

*Abbreviations*: *AMD* age-related macular degeneration, *BRVO* branch retinal vein occlusion, *CRVO* central retinal vein occlusion, *CME* cystoid macular edema, *CNVM* choroidal neovascular membrane, *DME* diabetic macular edema, *ERM* epi-retinal membrane, *FA* fluocinolone acetonide, *IRU* immune recovery uveitis, *IgG* immunoglobulin G, *CMV* cytomegalovirus, *NR* not reported, *PCR* polymerase chain reaction, *CD* cluster designation, *NK* natural killer, *N/A* not applicable

^a^Zone definitions are as follows: zone I defined as macula or optic nerve involvement; zone II defined as mid-periphery; and zone 3 defined as outer periphery. Zone definitions referenced in this paper: Cunningham ET Jr, Hubbard LD, Danis RP, Holland GN. Proportionate topographic areas of retinal zones 1, 2, and 3 for use in describing infectious retinitis. Arch Ophthalmol. 2011;129(11):1507-8 [115]

^b^Dosing with each modality varied widely across studies

**Table 5:** Summary of cases of CMV retinitis in the literature without human immunodeficiency virus infection

	CMV retinitis following intraocular and periocular corticosteroid injection^a^	CMV retinitis in the setting of immunodeficiency associated with thymoma (Good syndrome)	CMV retinitis in immunocompetent adults (non-Good syndrome)
Number of cases	*n* = 21, *n* = 21 eyes	*n* = 9, *n* = 10 eyes	*n* = 178, *n* = 248 eyes
Age (years)	Mean, 66.4 years	Mean, 56 years	Mean, 45.7 years
	Median, 69 years	Median, 56 years	Median, 48.0 year
	Range, 30.0–84.0 year	Range, 48–68 years	Range, 1 week–84 years
Gender	Male, 14/21 (66.6 %)	Male, 4/9 (44.4 %)	Male, 113/173 reported (65.3 %)
	Female, 7/21 (33.3 %)	Female, 5/9 (55.5 %)	Female, 60/173 reported (34.7 %)
	Male to female ratio, 2:1	Male to female ratio, 0.8:1	Male to female ratio, 1.88:1
% unilateral	21/21 (100 %)	8/9 (88.9 %)	108/178 (60.7 %) unilateral
Indication for corticosteroid	RVO, 7/21 (33.3 %)	N/A	N/A
	DME, 8/21 (38.0 %)		
	Uveitic CME, 7/21 (33.3 %)		
	CNVM due to AMD, 2/21 (9.5 %)		
	IRU, 0/21 (0.0 %)		
Corticosteroid dose/route	1.5–4 mg IVT, 8/21 (38.0 %)	N/A	N/A
	8–20 mg, 5/21 (23.8 %)		
	40 mg, 2/21 (9.0 %)		
	FA implant, 2/21 (9.0 %)		
	Range, 1.5–40 mg		
Time from corticosteroid dosing to retinitis (months)	Mean, 4.3 months	N/A	N/A
	Median, 4.0 months		
	Range, 0.25–13.0 months		
Timing of CMV retinitis relative to thymoma diagnosis (months)^b^	N/A	Retinitis diagnosed after thymoma, 8/9 (88.9 %)	N/A
		Mean, 31.4 months after thymoma	
		Median, 24 months after thymoma	
		Range, 75 months after to 1 month before	
Associated systemic diseases^b^	N/A	Respiratory infections, 7/9 (77.7 %)	No underlying systemic illness, 9/178 (5.1 %)
		Non-ocular CMV, 2/9 (22.2 %)	Organ or bone marrow transplant, 61/178 (34.3 %)
		Other opportunistic infections, 3/9 (33.3 %)	Autoimmune disease, 34/178 (19.1 %)
			Leukemia or lymphoma, 51/178 (28.7 %)
			Primary immune deficiency, 10/178 (5.6 %)
			Other systemic medical conditions, 24/178 (13.5 %)
Immunosuppressive medication	N/A	N/A	No medication, 55/160 reported (34.4 %)
			Using medication, 105/160 reported (65.6 %)
			Using multiple immunosuppressive medication, 78/105 (74.3 %)
			Using chemotherapy, 51/105 (48.6 %)
			Using antimetabolites or leukocyte signaling inhibitors, 49/105 (46.7 %)
Associated autoimmune diseases	N/A	Myasthenia gravis, 2/8 reported (25.0 %)	N/A
		Pure red cell aplasia, 2/9 cases (22.2 %)	
Associated immunologic laboratory abnormalities	N/A	Generalized lymphopenia: 3/5 reported (60.0 %)	N/A
		Low CD3+ T cells (<672 /mL), 1/3 reported (33.3 %)	
		Low CD4+ T cells (<360/μL), 5/6 reported (83.3 %)	
		Low CD 8 count (<240 /μL), 0/4 reported (0.0 %)	
		Low CD4+/CD8+ ratio (<0.6), 5/6 reported (83.3 %)	
		Low NK cells (<130 /mL), 1/2 reported (50.0 %)	
		Low serum IgG (<8 g/L), 7/8 reported (87.5 %)	
		Low serum IgM (<0.5 g/L), 7/8 reported (87.5 %)	
		Low serum IgA (<1.1 g/L), 8/8 reported (87.5 %)	
		Panhypogammaglobulinemia, 7/8 reported (87.5 %)	
CMV testing	Positive aqueous or vitreous PCR, 20/21 (95.2 %)	Positive aqueous PCR, 4/9 (44.4 %)	Positive aqueous PCR, 65/131 reported (49.6 %)
	Confirmed by other means, 1/21 (4.8 %)	Positive vitreous PCR, 5/9 (55.5 %)	Positive vitreous PCR, 29/131 reported (22.1 %)
		Confirmed by other means, 1/9 (11.1 %)	Confirmed by other means, 37/131 reported (28.2 %)
Zone involved^c^	Zone I, 4/20 reported (20.0 %)	Zone I, 5/8 reported (62.5 %)	Zone I, 72/97 eyes reported (74.2 %)
	Zone II, 17/20 reported (85.0 %)	Zone II, 4/8 reported (50 %)	Zone II, 87/97 eyes reported (89.7 %)
	Zone III, 1/20 reported (5.0 %)	Zone III, 2/8 reported (25 %)	Zone III, 39/97 eyes reported (40.2 %)
Vision when retinitis was first diagnosed	Acuity better than 20/40, 2/21 eyes (9.5 %)	Acuity better than 20/40, 0/9 eyes (0.0 %)	Acuity better than 20/40, 61/179 reported eyes (34.1 %)
	Acuity between 20/40 and 20/200, 11/21 eyes (52.3 %)	Acuity between 20/40 and 20/200, 7/9 eyes (77.7 %)	Acuity between 20/40 and 20/200, 70/179 reported eyes (39.1 %)
	Acuity worse than 20/200, 8/21 eyes (38.0 %)	Acuity worse than 20/200, 2/9 eyes (22.2 %)	Acuity worse than 20/200, 48/179 reported eyes (26.8 %)
Retinitis treatment^d^	Intravitreal therapy alone, 5/21 (23.8 %)	Intravitreal therapy alone, 1/9 (11.1 %)	Intravitreal therapy alone, 30/126 reported (23.8 %)
	Systemic therapy alone, 5/21 (23.8 %)	Systemic therapy alone, 2/9 (22.2 %)	Systemic therapy alone, 57/126 reported (45.2 %)
	Intravitreal and systemic therapy, 12/21 (52.3 %)	Intravitreal and systemic therapy, 6/9 (66.6 %)	Intravitreal and systemic therapy, 39/126 reported (31.0 %)
Follow-up (months)	Mean = 11.8 months	Mean = 4.56 months	Mean = 14.2 months
	Median = 5.5 months	Median = 6 months	Median = 6.0 months
	Range = 1–84 months	Range = 1.5–7 months	Range = 0–216 months
Vision at the last visit	Acuity better than 20/40, 2/21 eyes (9.5 %)	Acuity better than 20/40, 0/9 eyes (0.0 %)	Acuity better than 20/40, 52/171 reported eyes (30.4 %)
	Acuity between 20/40 and 20/200, 8/21 eyes (38.0 %)	Acuity between 20/40 and 20/200, 5/9 eyes (55.5 %)	Acuity between 20/40 and 20/200,:64/171 reported eyes (37.4 %)
	Acuity worse than 20/200, 11/21 eyes (52.3 %)	Acuity worse than 20/200, 4/9 eyes (44.4 %)	Acuity worse than 20/200, 55/171 reported eyes (32.2 %)

*Abbreviations*: *AMD* age-related macular degeneration, *BRVO* branch retinal vein occlusion, *CRVO* central retinal vein occlusion, *CME* cystoid macular edema, *CNVM* choroidal neovascular membrane, *DME* diabetic macular edema, *ERM* epiretinal membrane, *FA* fluocinolone acetonide, *IRU* immune recovery uveitis, *IgG* immunoglobulin G, *CMV* cytomegalovirus, *NR* not reported, *PCR* polymerase chain reaction, *CD* cluster designation, *NK* natural killer, *N/A* not applicable

^a^Data used from paper done by Takakura et al. [89] currently in peer review “Ocular Immunology and Inflammation”

^b^All patients were HIV negative

^c^Zone definitions are as follows: Zone I defined as macula or optic nerve involvement; Zone II defined as mid-periphery; Zone 3 defined as outer periphery. Zone definitions referenced in this paper: Cunningham ET Jr, Hubbard LD, Danis RP, Holland GN. Proportionate topographic areas of retinal zones 1, 2, and 3 for use in describing infectious retinitis. Arch Ophthalmol. 2011;129(11):1507–8 [115]

^d^Dosing with each modality varied widely across studies

The most complete and standardized description of CMVR in HIV-positive patients comes from the studies performed by the Studies of the Ocular Complications of AIDS (SOCA) research group [101–103]. Cytomegalovirus retinitis was the most frequently encountered complication of HIV infection in this cohort, occurring in 34.6 % of patients with CD4+ T cell counts <50 cells/μL and in 63.4 % of patients with CD4+ T cell counts <200 cells/μL [104]. Clinically, HIV-associated CMVR includes classic features of necrotizing retinitis with irregular sheathing of adjacent vessels and variable degrees of hemorrhage (sometimes referred to as “pizza pie retinopathy” or “cottage cheese with ketchup”), and which is sometimes coupled with a frost branch angiitis appearance without vascular occlusion and often associated with mild vitreous or anterior chamber inflammation [12, 20, 104]. In addition to these core clinical findings previously listed, the SOCA studies quantified the prevalence of a number of hallmark features of CMVR at presentation. Specifically, keratic precipitates were present in 36.8 % of the eyes with CMVR, anterior chamber inflammation in 46.2 %, and vitreous inflammation in 61.9 %. The prevalence of anterior chamber cells greater than 2+ was 1.9 % and vitreous haze greater than 2+ was 11.4 %. Macular edema, epiretinal membrane formation, and rhegmatogenous retinal detachment were each uncommon at presentation in the SOCA cohort, occurring in less than 10 % of the eyes, and posterior synechiae formation was not observed [104].

A number of studies have suggested that the clinical presentation of CMVR in HIV-negative patients can differ from that in HIV-positive patients [20–23, 27, 36, 94]. In a small series of eyes with CMVR in HIV-negative patients complied by Maguire and associates in 1995, three eyes were found to have spontaneously regressed or indolent appearing CMVR associated with vitritis and CME. The authors suggested that HIV-negative CMVR was more often associated with moderate to severe vitreous inflammation and CME [21]. The tendency for HIV-negative CMVR to have more severe intraocular inflammation as compared to CMVR in HIV-positive patients has since been noted by a number of authors, including Silverstein and colleagues [23], Voros and associates [27], Tajunisah and colleagues [25], Panthanapitoon and associates [22], and Schneider and colleagues [36] with specific mention of a similarity to acute retinal necrosis (ARN) in some eyes, including both the severity of the inflammation and the presence of occlusive vasculitis. In most instances, the more severe inflammation and vascular occlusion was ascribed to relative retention of anti-CMV immunoreactivity. However, other studies have failed to identify consistent difference in clinical presentation based on HIV status [18–20, 24, 26, 30–33, 37–40, 44, 94, 95]. In our review, of all the reported cases to date of HIV-negative CMVR not associated with Good syndrome or following intraocular or periocular administration of corticosteroids, clinical features of the inflammation were reported in 199 of the 248 eyes (80.2 %). Within these 199 eyes, 13 (6.5 %) were specifically described as having moderate to severe anterior chamber inflammation, 24 (12.1 %) as having moderate to severe vitreous inflammation, and 47 (23.6 %) were noted to have occlusive vasculitis. In HIV-negative CMVR associated with Good syndrome, clinical features of the inflammation were reported in all eyes. Within these 10 eyes, only 1 (10.0 %) was specifically described as having moderate to severe anterior chamber inflammation, whereas 5 out of 10 eyes (50.0 %) were described as having moderate to severe vitreous inflammation. None were noted to have occlusive vasculitis. In HIV-negative CMVR following intraocular or periocular administration of corticosteroids, clinical features of the inflammation were reported in 12 of the 21 eyes (57.1 %). Among these 12 eyes, 4 (33.3 %) each were described as moderate to severe anterior chamber inflammation or vitreous inflammation, and 6 (50.0 %) were noted to have occlusive retinal vasculitis. Hence, while an ARN-like picture including moderate to severe intraocular inflammation and the presence of occlusive vasculitis may be somewhat more common in HIV-negative as compared to HIV-positive CMVR, particularly in the setting of Good syndrome or following periocular or intraocular corticosteroids, the clinical presentation in these various cohorts appears, more often than not, to be fairly similar, with an overlapping clinical presentation (Table 5).

Immune recovery uveitis has been well characterized in HIV-positive patients and is currently one of the most common causes of vision loss in patients with CMVR receiving highly active antiretroviral therapy (HAART) [104, 105]. While the occurrence of IRU has varied widely in HIV-positive cohorts receiving HAART, with reported rates ranging from 3.0 to 63.3 % [106], the inflammation generally occurs several weeks to months after initiating HAART, as the number of circulating CD4+ T cells increases. The clinical spectrum of IRU includes vitritis, papillitis, CME, epiretinal membrane formation, vitreous hemorrhage, retinal neovascularization, vitreomacular traction syndrome, and proliferative vitreoretinopathy [104–107].

Of the 248 eyes of 178 patients with CMVR in the absence of either HIV infection or Good syndrome that have been described in the literature (Additional file 1: Table S1), 16 eyes (6.5 %) of 10 patients had one or more features consistent with IRU [17, 20, 45, 50, 70]. Reported ages of these 10 patients ranged from 15 to 68 years, with a mean and median of 47.8 and 54 years, respectively. Men outnumbered women approximately two to one (M to F ratio = 2.3:1), and the 100 % of the cases had an identifiable cause of systemic immunosuppression. The most common factors contributing to a relative decline in immune function included an underlying malignancy (*n* = 3; 30.0 %), age over 60 years (*n* = 2; 20.0 %), an autoimmune disorder (*n* = 2; 20.0 %), organ (*n* = 3; 30.0 %) or bone marrow (*n* = 1; 10.0 %) transplantation requiring systemic immunosuppression, and diabetes mellitus (*n* = 1; 10.0 %). The three reported cancers included two patients with acute lymphoblastic leukemia and one patient with chronic lymphocytic leukemia. The use of systemic immunosuppressive medication was reported in 8 out of the 10 cases (80.0 %). All of these patients were on two or more immunosuppressive agents. Corticosteroids were the most common immunosuppressive agent used in four patients (50.0 %), followed by cyclophosphamide in three patients (37.5 %), and two patients each (25.0 %) treated with vincristine and mycophenolate mofetil. One patient each (12.5 %) was treated with rituximab, fludarabine, methotrexate, and tacrolimus. Clinically, the retinitis was bilateral in 6 (60.0 %) out of 10 cases. Most case reports provided limited clinical information, and the location was either reported or illustrated in only 1 out of the 10 patients (10.0 %). In this patient, the retinitis was found to involve zone II. Additional clinical features were noted in 5 out of 16 eyes (31.3 %). Among these five eyes, just two eyes (40.0 %) were noted to have moderate anterior chamber inflammation. All five eyes (100.0 %) were noted to have vitreous inflammation, among which inflammation was described moderate to severe in all cases. An occlusive vasculitis was not present in any reported case of CMVR associated with IRU. Visual acuity at initial CMVR diagnosis was reported in 15 out of 16 eyes (93.8 %). Among these 15 eyes, visual acuity at initial diagnosis was better than 20/40 in 40.0 %, between 20/40 and 20/200 in 53.3 %, and worse than 20/200 in 6.7 % of eyes. The method of diagnostic confirmation of CMVR was reported in 1 of the 10 cases (10.0 %), among which the diagnosis of CMVR was confirmed serum PCR testing. The retinitis responded to antiviral therapy in all cases. The therapeutic treatment administered was reported in 6 of the 10 cases (60.0 %). Of these 6 cases, treatment was administered systemically only in 33.3 % of patients and intravitreally alone in 66.7 % patients. Visual acuity at last follow-up visit was reported in 8 patients and 13 eyes (mean 22.2 months; median 19 months; range 1 to 43 months). Visual acuity at last follow-up was better than 20/40 in 38.5 %, between 20/40 and 20/200 in 46.1 %, and worse than 20/200 in 15.4 % of eyes.

Since the December, 2014, cutoff for our literature review, there have been several publications of CMVR in HIV-negative patients [108–113]. While these publications are not incorporated into this review, the clinical context in which CMVR developed and the clinical characteristics of the retinitis and the treatment(s) given for the infection were not significantly different from those previously reported or from the conclusions drawn by our review [114].

## Conclusions

Although uncommon, CMVR can occur in the absence of HIV infection. Over 95 %, of HIV-negative patients who developed CMVR were found, ultimately, to have one or more factors contributing to a relative decline in immune function, such as advanced age, an underlying malignancy, an autoimmune disease or organ/bone transplantation requiring systemic immunosuppression, administration of periocular or intraocular corticosteroids, diabetes mellitus, or, less commonly, an inherited or acquired immune disorder, such as Good syndrome. While the clinical features of CMVR were generally similar in HIV-negative and HIV-positive patients, accumulated data regarding the rate of moderate to severe intraocular inflammation and occlusive retinal vasculitis would seem to suggest that these more ARN-like features occur more often in HIV-negative patients.

## Additional file

Additional file 1: Table S1. Summary of reported cases of cytomegalovirus (CMV) retinitis in immunocompetent HIV-negative patients. (DOC 497 kb)
